# Association of Environmental tobacco smoke exposure with depression among non-smoking adults

**DOI:** 10.1186/s12889-021-11780-y

**Published:** 2021-09-26

**Authors:** Akinkunmi Paul Okekunle, Jeffery Osahon Asowata, Jung Eun Lee, Onoja Matthew Akpa

**Affiliations:** 1 Department of Epidemiology and Medical Statistics, College of Medicine, University of Ibadan, Post Office200284 PMB, Ibadan, UI 900001 Nigeria; 2 The Postgraduate College, University of Ibadan, Ibadan, 200284 Nigeria; 3 Department of Food and Nutrition, College of Human Ecology, Seoul National University, 1 Gwanak-ro, Gwanak-gu, Seoul, IL 08826 South Korea; 4 Research Institute of Human Ecology, Seoul National University, 1 Gwanak-ro, Gwanak-gu, Seoul, IL 08826 South Korea; 5 Center for Genomic and Precision Medicine, College of Medicine, University of Ibadan, Ibadan, 200284 Nigeria; 6 Preventive Cardiology Research Unit, Institute of Cardiovascular Diseases, College of Medicine, University of Ibadan, Ibadan, 200284 Nigeria

**Keywords:** ETSE, Depression, Mental health, NHANES

## Abstract

**Background:**

Depression is a psychological dysfunction that impairs health and quality of life. However, whether environmental tobacco smoke exposure (ETSE) is associated with depression is poorly understood. This study was designed to evaluate the association of ETSE with depression among non-smoking adults in the United States.

**Method:**

Using the 2015–2016 United States National Health and Nutrition Examination Survey (NHANES), we identified 2623 adults (females – 64.2%, males – 35.8%) who had never smoked and applied multivariable adjusted-logistic regression to determine the adjusted odds ratio (aOR) and 95% confidence interval (CI) at *P* < 0.05 for the association of ETSE with depression adjusting for relevant confounders.

**Results:**

Mean age of respondents was 46.5 ± 17.9 years, 23.5% reported ETSE, and 4.7% reported depression. Also, aORs for the association of ETSE with depression were 1.992 (1.987, 1.997) among females and 0.674 (0.670, 0.677) among males. When we examined the association by age groups, the aORs were 1.792 (1.787, 1.796) among young adults (< 60 years) and 1.146 (1.140, 1.152) among older adults (≥60 years).

**Conclusions:**

We found that ETSE was associated with higher odds of depression among females but not among males.

## Background

Environmental tobacco smoke exposure (ETSE) is the exposure to smoke arising from the burning of any form of tobacco product(s) or exhalation by a person who smokes any form of tobacco product [1, 2]. Several pieces of evidence suggest that ETSE may be a major modifiable risk factor for morbidity and mortality globally [3, 4]. Also, ETSE has been suggested to be associated with productivity losses [5] and responsible for 600,000 deaths per year in the United States (US) [3, 6]. A recent report revealed a decline in the prevalence of tobacco smoking among males and females [7] without itemizing ETSE rates and implications on mental health. Depression is a widespread mood disorder [8, 9] associated with morbidity and mortality worldwide [5]. It affects one out of every five people in a lifetime and is one of the leading causes of disability worldwide [10]. It is characterized by symptoms associated with imbalance(s) in emotional, motivational, cognitive, and physiological wellbeing [10, 11].

Several studies have reported the relationship between smoking and depressive symptoms [8, 12–17], which prompted legislative interventions [18, 19] to minimize smoking rates. However, ETSE is an evolving phenomenon worldwide with the potential of making vulnerable populations at odds of adverse health outcomes [20–24]. Some animal studies [25–29] have reported the potential inhibitory effect of ETSE on dopamine transport and metabolism. Dopamine is a neurotransmitter with multiple functions in neurological processes and mental health [30]. For example, dopamine-receptor localization attributable to ETSE [27] has been linked with psychologically-related deformities in rat models [28]. Whether similar exposures can affect mental health in human populations is yet to be clearly understood.

It is not yet clear whether ETSE is associated with alteration(s) that could promote depressive symptoms and/or disorders in the neural network among humans. Understanding the importance of ETSE in depression outcomes could offer useful new information to guide the design of well-articulated public health policies, guidelines or advisory for the effective management of ETSE to avoid depressive symptoms and adverse health conditions.

The current analysis examined the association of ETSE with depression among non-smokers in the National Health and Nutrition Examination Survey (NHANES). We hypothesized ETSE had a null association with depression and assessed whether the association differed by sex and age in the same population.

## Methods

### Study design and sampling strategies

We used the 2015–2016 NHANES survey data by the National Center for Health Statistics of the Centers for Disease Control and Prevention (CDC) in the US for this study [31]. Using a multistage probability technique, 15,327 non-institutionalized civilian residents of the United States across 50 states and Washington DC were sampled, and 9971 completed the interview. Specific subgroups populations were factored into the sampling to increase the consistency and accuracy of estimates in the US population [32].

**Fig. 1:**
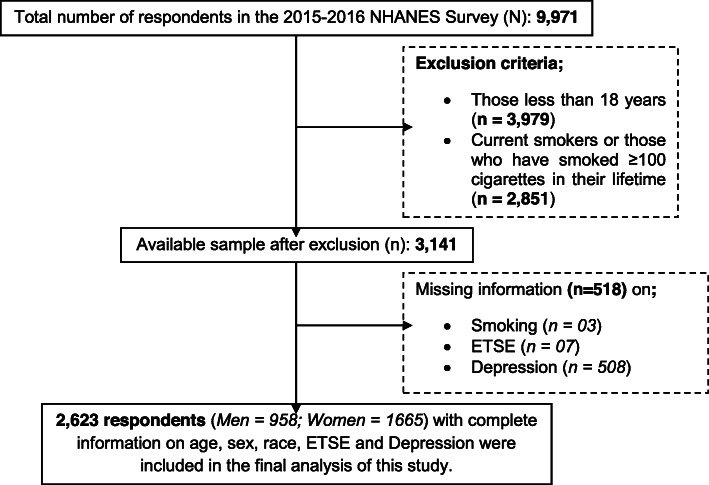
Flowchart describing selection of respondents for this study

From the 9971 respondents that completed the interview, we excluded respondents less than 18 years (3979) and active smokers (2851), i.e. those who reported they had smoked at least 100-lifetime cigarettes or currently smoke/consume any form of tobacco products or e-cigarette. Also, 518 respondents with missing data (smoking, ETSE and depression status) were excluded from this current analysis. In all, 2623 respondents (958 males and 1665 females) were included in the final analysis of this report. A detailed description of how respondents were selected for the final analysis in this study is presented in Fig. 1.

### Definition of depression (outcome)

Using the patient health questionnaire (PHQ-9) depression scale, respondents provided information on experience(s) and frequency of depressive symptoms in the last two weeks preceding the survey. The PHQ-9 is a 9-item scale designed to probe information on interest or pleasure in doing things, feeling down or hopeless, sleeping problems, etc. Respondents reported whether and how often they experienced depressive symptoms such as; little interest or pleasure in doing things, feeling down or hopeless, sleeping problems, feeling of tiredness or having little energy, etc. Responses to each item on the PHQ-9 scale ranged from ‘*not at all*’, ‘*several days*’, ‘*more than half the day*’ to ‘*nearly every day*’ with a consecutive score of 0, + 1, + 2 and + 3, respectively. Overall depression score was computed by aggregating the score assigned to each item on the PHQ-9 scale. Details of the criteria for assessment, scoring guide, validation, reliability and efficiency of PHQ-9 has been reported elsewhere [33]. A respondent is classified to be likely undergoing depression if the PHQ-9 score ≥ 10 according to the PHQ-9 scoring guide [33] and some previously published studies [34, 35].

### Environmental tobacco smoke exposure – ETSE (exposure)

In the NHANES data, respondents were requested to provide information on whether they had worked at a job or spent time in a restaurant/bar/car/indoor area where someone smoked indoors (at least once) in the last seven days. ETSE was defined as a self-reported exposure to smoke arising from burning any form of tobacco products or exhaling by a smoker [1, 2] in any indoor environment.

### Data collection and variables of the study (confounding)

Trained staff collected information on demographic and lifestyle characteristics through in-person interviews using validated survey instruments. Demographic characteristics include; sex (*male/female*), age in years, and race (Hispanics only/White only/Black only/Others). Education was defined as *≥High School if the respondent reported completing* formal education (at least 9th grade) otherwise *<High School*. Employment status was defined as ‘yes’ the respondent currently engaged in any form of a paid job, otherwise ‘no’. Annual household income was dichotomized as *≤ $24,999 or > $24,999.* Marital status was defined as never married, married/living with a partner and widowed/divorced/separated. Alcohol use was defined as ‘yes’ if the respondent took at least 12 drinks of alcoholic drink in the past year or a lifetime, otherwise ‘no’.

### Statistical analysis

All statistical analyses were computed at a statistical significance of two-sided *P* < 0.05 using IBM SPSS Statistics for Windows, version 21 (IBM Corporation, Armonk, NY USA). Respondents’ characteristics were stratified by depression status (no/yes) and compared using Chi-square (*χ*2) test or independent sample *t-*test for categorical or continuous data, respectively. Multivariable adjusted logistic regression was applied to estimate the adjusted odds ratio (aOR) and 95% confidence interval (CI) for the association of ETSE with depression (adjusting for socio-demographic and lifestyle factors) in the overall sample. We stratified the analysis of the ETSE – depression association by sex (male/female), age groups (younger adults; < 60 years and older adults; ≥60 years), race (Hispanics only, Whites only, Blacks only and Others), employment status (no, yes), annual household income (no, yes), marital status (never married, married and widowed) and alcohol use (no, yes). Also, a likelihood ratio test was carried out to test the significance of the interaction of demographic and lifestyle factors with ETSE in the logistic regression models. All estimates in this report were weighted (using the Centers for Disease Control and Prevention guidelines) to reduce potential biases attributable to complex sample designs and unequal probabilities in sampling and calibrated to the overall US population (to minimize coverage disparities and reduce variances in estimation techniques) [32].

## Results

### Characteristics of non-smoking adults in the 2015–2016 NHANES survey data

Characteristics of respondents are presented in Table 1. Overall, the mean age was 46.5 ± 17.9 years, and 63.4% of respondents in this study were females. Also, 58.8% of the respondents were Whites, 3.5% had at least a high school education and more than half (65.3%) were currently employed. The majority (83.5%) of the respondents had an annual household income of more than $24,999, and 17.4% were never married. Similar distributions were observed for the age-stratified analyses (Tables 2 & 3), but 86.4% and 30.0% of young and old adults, respectively, were employed. The proportions of widowed respondents were 15.5%, 8.2% and 36.0% in the overall sample, among young and old adults, respectively.

**Table 1:** Characteristics of all respondents according to Depression status in the 2015–2016 NHANES data

Characteristics	All Respondents	Depression Status^†^ (*N* = 2623)		*P*
		No	Yes	
Sex				
*Male*	958 (36.6)	922 (97.1)	36 (2.9)	< 0.0001
*Female*	1665 (63.4)	1555 (94.3)	110 (5.7)	
Age (years)	46.5 ± 17.9	46.4 ± 17.9	47.8 ± 17.7	< 0.0001
*< 60 years*	1887 (74.5)	1789 (95.4)	98 (4.6)	< 0.0001
*≥ 60 years*	736 (25.5)	688 (95.0)	48 (5.0)	
Race				
*Hispanics only*	911 (18.2)	846 (93.7)	65 (6.3)	< 0.0001
*White only*	686 (58.8)	654 (96.0)	32 (4.0)	
*Black only*	554 (12.4)	527 (95.5)	27 (4.5)	
Others	472 (10.6)	450 (94.2)	22 (5.8)	
Education				
*< High School*	2459 (96.4)	2325 (95.3)	134 (4.7)	< 0.0001
*≥ High School*	162 (3.5)	150 (95.3)	12 (4.7)	
Employed				
*No*	1056 (34.7)	961 (92.3)	95 (7.7)	< 0.0001
*Yes*	1559 (65.3)	1508 (96.9)	51 (3.1)	
Annual Household Income				
*≤ $24,999*	631 (16.5)	565 (88.6)	66 (11.4)	< 0.0001
*> $24,999*	1806 (83.5)	1735 (96.7)	71 (3.3)	
Marital Status				
*Never married*	448 (17.4)	405 (92.0)	43 (8.0)	< 0.0001
Married*	1534 (67.1)	1479 (96.9)	55 (3.1)	
*Widowed*^*#*^	478 (15.5)	441 (92.2)	37 (7.8)	
Alcohol use				
*No*	835 (24.8)	786 (95.4)	49 (4.6)	< 0.0001
*Yes*	1784 (75.2)	1687 (95.3)	97 (4.7)	

† − Depression status was defined using the PHQ-9 depression scale

* - married/living with partner, # - widowed/divorced/separated

Continuous variables are presented as mean ± standard deviation and compared using the t-test

Categorical variables are presented as n(%) and compared using the *x*^2^ test

**Table 2:** Characteristics of respondents < 60 years only according to Depression status in the 2015–2016 NHANES data

Characteristics	< 60 years only	Depression Status^†^ (*n* = 1887)		*P*
		No	Yes	
Sex				
*Male*	715 (38.7)	691 (97.0)	24 (3.0)	< 0.0001
*Female*	1172 (61.3)	1098 (94.5)	74 (5.5)	
Age (years)	38.2 ± 12.2	38.2 ± 12.2	39.6 ± 13.1	< 0.0001
Race				
*Hispanics only*	657 (21.11)	617 (94.3)	40 (5.7)	< 0.0001
*White only*	409 (53.2)	392 (96.3)	17 (3.7)	
*Black only*	436 (14.0)	416 (95.7)	20 (4.3)	
Others	385 (11.7)	364 (93.2)	21 (6.8)	
Education				
*< High School*	1724 (95.3)	1637 (95.4)	87 (4.6)	< 0.0001
*≥ High School*	161 (4.7)	150 (95.5)	11 (4.5)	
Employed				
*No*	522 (22.7)	469 (91.0)	53 (9.0)	< 0.0001
*Yes*	1329 (77.3)	1314 (96.7)	45 (3.3)	
Annual Household Income				
*≤ $24,999*	367 (13.6)	332 (91.0)	35 (9.0)	< 0.0001
*> $24,999*	1767 (86.4)	1340 (96.1)	60 (3.9)	
Marital Status				
*Never married*	404 (21.9)	365 (91.6)	39 (8.4)	< 0.0001
Married*	1137 (69.9)	1101 (97.1)	36 (2.9)	
*Widowed*^*#*^	183 (8.2)	171 (91.2)	12 (8.8)	
Alcohol use				
*No*	558 (23.7)	531 (95.6)	27 (4.4)	< 0.0001
*Yes*	1328 (76.3)	1257 (95.4)	71 (4.6)	

† − Depression status was defined using the PHQ-9 depression scale

* - married/living with partner, # - widowed/divorced/separated

Continuous variables are presented as mean ± standard deviation and compared using the t-test

Categorical variables are presented as n(%) and compared using the *x*^2^ test

**Table 3:** Characteristics of respondents ≥60 years only according to Depression status in the 2015–2016 NHANES data

Characteristics	≥ 60 years only	Depression Status^†^ (*n* = 736)		*P*
		No	Yes	
Sex				
*Male*	243 (30.3)	231 (97.6)	12 (2.4)	< 0.0001
*Female*	493 (69.7)	457 (93.8)	36 (6.2)	
Age (years)	70.7 ± 6.5	70.7 ± 6.4	69.5 ± 6.6	< 0.0001
Race				
*Hispanics only*	254 (9.7)	229 (90.1)	25 (9.9)	< 0.0001
*White only*	277 (75.1)	262 (95.3)	15 (4.7)	
*Black only*	118 (7.7)	111 (94.6)	07 (5.4)	
Others	87 (7.5)	86 (98.7)	01 (1.3)	
Education				
*< High School*	735 (99.9)	688 (95.0)	47 (5.0)	< 0.0001
*≥ High School*	01 (0.1)	00 (0.0)	01 (100.0)	
Employed				
*No*	534 (70.0)	492 (93.6)	42 (6.4)	< 0.0001
*Yes*	200 (30.0)	194 (98.1)	06 (1.9)	
Annual Household Income				
*≤ $24,999*	264 (25.3)	233 (84.6)	31 (15.4)	< 0.0001
*> $24,999*	406 (74.7)	395 (98.7)	11 (1.3)	
Marital Status				
*Never married*	44 (4.8)	40 (97.5)	04 (2.5)	< 0.0001
Married*	397 (59.2)	378 (96.1)	19 (3.9)	
*Widowed*^*#*^	295 (36.0)	270 (92.8)	25 (7.2)	
Alcohol use				
*No*	277 (28.1)	255 (94.8)	22 (5.2)	< 0.0001
*Yes*	456 (71.9)	430 (95.0)	26 (5.0)	

† − Depression status was defined using the PHQ-9 depression scale

* - married/living with partner, # - widowed/divorced/separated

Continuous variables are presented as mean ± standard deviation and compared using the t-test

Categorical variables are presented as n(%) and compared using the *x*^2^ test

### Prevalence of ETSE among non-smoking adults in the 2015–2016 NHANES survey data

**Fig. 2:**
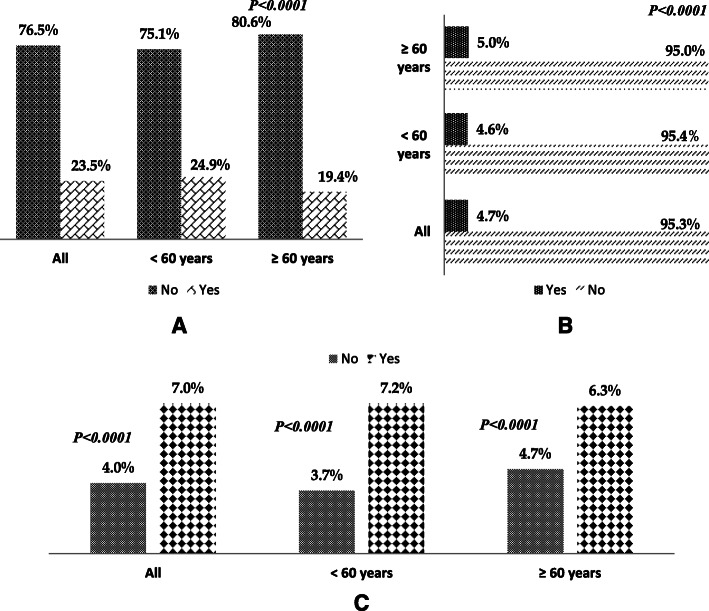
Distribution of ETSE *(no/yes)* (**A**), depression *(no/yes)* (**B**), depressed respondents stratified by ETSE (no/yes) (**C**) among non-smokers only. *P*-values were calculated based on the chi-square test

Overall, 23.5% of the entire sample reported ETSE (Fig. 2A). Also, the proportion of respondents who reported ETSE among young adults (24.9%) was significantly higher (*P < 0.0001*) than their counterparts among old adults (19.4%).

### Prevalence of and factors associated with depression among non-smoking adults in the 2015–2016 NHANES survey data

Overall, 4.7% of the entire sample reported having depression (Fig. 2B), with a significantly higher proportion among old adults (5.0%), females (5.7%), among those unemployed (7.7%), among low-income households (11.4%) and alcohol users (4.7%) – Table 1. Age-stratified analysis revealed a similar trend predominantly among young adults.

Furthermore, females had 1.9 times higher odds of being depressed; aOR: 1.898 (1.893, 1.903) compared to males (Table 4). Also, Whites only; aOR: 0.764 (0.762, 0.767) and Blacks only; aOR: 0.643 (0.641, 0.645) had lesser odds of being depressed compared to Hispanics only. Similarly, those who were employed (compared to those unemployed); aOR: 0.479 (0.478, 0.480) and respondents from households with income *greater than $24,999* (compared with those from households with an annual income of *less than $24,999*); aOR: 0.361 (0.360, 0.362) had lesser odds of being depressed. Similar trends were observed across sex and age groups (Tables 5, 6 & 7), but Whites only; aOR: 3.657 (3.606, 3.708) and Blacks only; aOR: 5.503 (5.429, 5.579) had higher odds of being depressed compared to Hispanics only among old adults. Also, respondents who are married; aOR: 7.328 (7.225, 7.434) and widowed; aOR: 11.730 (11.561, 11.901) had higher odds of being depressed compared to those who were never married among old adults. Those who reported alcohol use had 1.7 times higher odds of being depressed; aOR: 1.733 (1.729, 1.737) than those who do not take alcohol. The results were largely unaltered after stratifying the analyses by sex (Table 5).

**Table 4:** ETSE and odds of Depression in the 2015–2016 NHANES data

Factors	All		< 60 years		≥60 years	
	aOR (95%CI)	*P*	aOR (95%CI)	*P*	aOR (95%CI)	*P*
*ETSE*						
(*Yes*)	1.625 (1.622, 1.629)	< 0.0001	1.792 (1.787, 1.796)	< 0.0001	1.066 (1.060, 1.071)	< 0.0001
Sex						
(*Female*)	1.898 (1.893, 1.903)	< 0.0001	1.845 (1.840, 1.850)	< 0.0001	1.916 (1.905, 1928)	< 0.0001
Age (years)	0.996 (0.996, 0.996)	< 0.0001	1.030 (1.030, 1.030)	< 0.0001	0.863 (0.863, 0.863)	< 0.0001
Race*						
*(White only)*	0.764 (0.762, 0.767)	< 0.0001	0.667 (0.664, 0.669)	< 0.0001	3.657 (3.606, 3.708)	< 0.0001
*(Black only)*	0.643 (0.641, 0.645)	< 0.0001	0.558 (0.556, 0.560)	< 0.0001	5.503 (5.429, 5.579)	< 0.0001
*(Others)*	0.461 (0.459, 0.462)	< 0.0001	0.418 (0.416, 0.419)	< 0.0001	1.345 (1.324, 1.366)	< 0.0001
Employed						
(*Yes*)	0.479 (0.478, 0.480)	< 0.0001	0.388 (0.387, 0.389)	< 0.0001	0.177 (0.176, 0.178)	< 0.0001
Annual Household Income (*>$24,999*)	0.361 (0.360, 0.362)	< 0.0001	0.718 (0.716, 0.720)	< 0.0001	0.049 (0.049, 0.050)	< 0.0001
Marital Status						
(*Married*)^#^	0.414 (0.413, 0.415)	< 0.0001	0.236 (0.235, 0.236)	< 0.0001	7.328 (7.225, 7.434)	< 0.0001
(Widowed)	0.750 (0.747, 0.752)	< 0.0001	0.572 (0.569, 0.574)	< 0.0001	11.730 (11.561, 11.901)	< 0.0001
Alcohol use						
(*Yes*)	1.733 (1.729, 1.737)	< 0.0001	1.422 (1.417, 1.426)	< 0.0001	2.326 (2.314, 2.338)	< 0.0001

Model was adjusted for sex (‘male’ as reference), age (continuous in years), race (‘Hispanics’ as reference), employment (‘no’ as reference), annual household income (*≤ $24,999 as reference*) marital status (*never married/single as reference*) and alcohol use (no as reference)

**Table 5:** ETSE and odds of Depression in the 2015–2016 NHANES data stratified by sex

Characteristics	All respondents			
	Male		Female	
	aOR (95%CI)	*P*	aOR (95%CI)	*P*
*ETSE* (*Yes*)	0.674 (0.670, 0.677)	< 0.0001	1.992 (1.987, 1.997)	< 0.0001
Age (years)	0.991 (0.991, 0.991)	< 0.0001	0.998 (0.998, 0.998)	< 0.0001
Race*				
*(White only)*	1.100 (1.092, 1.108)	< 0.0001	0.709 (0.706, 0.712)	< 0.0001
*(Black only)*	0.837 (0.831, 0.842)	< 0.0001	0.591 (0.589, 0.593)	< 0.0001
*(Others)*	0.596 (0.591, 0.601)	< 0.0001	0.439 (0.437, 0.441)	< 0.0001
Employed				
(*Yes*)	0.356 (0.354, 0.357)	< 0.0001	0.526 (0.525, 0.527)	< 0.0001
Annual Household Income (*>$24,999*)	0.461 (0.459, 0.464)	< 0.0001	0.340 (0.339, 0.341)	< 0.0001
Marital Status^#^				
(*Married*)	0.350 (0.348, 0.352)	< 0.0001	0.445 (0.444, 0.447)	< 0.0001
(Widowed)	1.409 (1.399, 1.419)	< 0.0001	0.672 (0.669, 674)	< 0.0001
Alcohol use (*Yes*)	2.875 (2.855, 2.895)	< 0.0001	1.618 (1.613, 1.622)	< 0.0001

Model was adjusted for age (continuous in years), race (‘Hispanics’ as reference), employment (‘no’ as reference), annual household income (*≤ $24,999 as reference*) marital status (*never married/single as reference*) and alcohol use (no as reference)

**Table 6:** ETSE and odds of Depression in the 2015–2016 NHANES data stratified by sex among those < 60 years only

Characteristics	< 60 years only			
	Male		Female	
	aOR (95%CI)	*P*	aOR (95%CI)	*P*
*ETSE* (*Yes*)	0.713 (0.708, 0.717)	< 0.0001	2.334 (2.327, 2.340)	< 0.0001
Age (years)	1.035 (1.035, 1.035)	< 0.0001	1.030 (1.030, 1.030)	< 0.0001
Race*				
*(White only)*	0.822 (0.816, 0.829)	< 0.0001	0.647 (0.644, 0.650)	< 0.0001
*(Black only)*	0.849 (0.843, 0.855)	< 0.0001	0.491 (0.489, 0.493)	< 0.0001
*(Others)*	0.346 (0.342, 0.349)	< 0.0001	0.443 (0.440, 0.445)	< 0.0001
Employed				
(*Yes*)	0.226 (0.225, 0.227)	< 0.0001	0.446 (0.445, 0.448)	< 0.0001
Annual Household Income (*>$24,999*)	0.531 (0.527, 0.534)	< 0.0001	0.842 (0.839, 0.845)	< 0.0001
Marital Status^#^				
(*Married*)	0.228 (0.227, 0.230)	< 0.0001	0.245 (0.244, 0.245)	< 0.0001
(Widowed)	0.831 (0.824, 0.838)	< 0.0001	0.465 (0.462, 0.467)	< 0.0001
Alcohol use (*Yes*)	7.297 (7.225, 7.369)	< 0.0001	1.076 (1.072, 1.079)	< 0.0001

Model was adjusted for age (continuous in years), race (‘Hispanics’ as reference), employment (‘no’ as reference), annual household income (*≤ $24,999 as reference*) marital status (*never married/single as reference*) and alcohol use (no as reference)

**Table 7:** ETSE and odds of Depression in the 2015–2016 NHANES data stratified by sex among those ≥60 years only

Characteristics	≥ 60 years only			
	Male		Female	
	aOR (95%CI)	*P*	aOR (95%CI)	*P*
*ETSE* (*Yes*)	0.413 (0.406, 0.421)	< 0.0001	1.207 (1.200, 1.214)	< 0.0001
Age (years)	0.874 (0.873, 0.875)	< 0.0001	0.856 (0.855, 0.856)	< 0.0001
Race*				
*(White only)*	†	< 0.0001	2.531 (2.495, 2.568)	< 0.0001
*(Black only)*	†	< 0.0001	4.766 (4.700, 4.833)	< 0.0001
*(Others)*	†	< 0.0001	0.482 (0.473, 0.491)	< 0.0001
Employed				
(*Yes*)	0.477 (0.470, 0.483)	< 0.0001	0.106 (0.105, 0.107)	< 0.0001
Annual Household Income (*>$24,999*)	0.234 (0.231, 0.237)	< 0.0001	0.033 (0.032, 0.033)	< 0.0001
Marital Status^#^				
(*Married*)	3.113 (3.038, 3.191)	< 0.0001	8.639 (8.483, 8.798)	< 0.0001
(Widowed)	8.856 (8.632, 9.086)	< 0.0001	13.117 (12.880, 13.359)	< 0.0001
Alcohol use (*Yes*)	0.645 (0.637, 0.654)	< 0.0001	2.237 (2.224, 2.251)	< 0.0001

Model was adjusted for age (continuous in years), race (‘Hispanics’ as reference), employment (‘no’ as reference), annual household income (*≤ $24,999 as reference*) marital status (*never married/single as reference*) and alcohol use (no as reference)

† - estimation was exempted due to insufficient sample size

### ETSE and depression among non-smoking adults in the 2015–2016 NHANES survey data

Overall, the prevalence of depression among respondents with ETSE (7.0%) was significantly higher compared to those without ETSE (4.0%) (Fig. 2C). A similar trend was observed after stratifying by age.

Overall (Table 4), respondents exposed to ETSE had 1.6 times higher odds of being depressed; aOR: 1.625 (1.622, 1.629) compared to those unexposed to ETSE in the overall sample. Similarly, young adults exposed to ETSE had 1.7 times higher odds of being depressed; aOR: 1.792 (1.787, 1.796) compared to similar respondents unexposed to ETSE.

### Subgroup analyses of the association of ETSE with depression

The association of ETSE with depression was stratified by sex, age groups, race, employment status, annual household income and alcohol use (Table 8), and remained independent of age groups, employment status, annual household income and alcohol use. However, aORs for the association of ETSE with depression was 1.992 (1.987, 1.997) among females and 0.674, (0.670, 0.677) among males P for interaction < 0.0001. Also, aORs for the association of ETSE with depression was aOR: 0.552 (0.549, 0.556) among Blacks only, 1.410 (1.403, 1.417) among Hispanics only and 1.646 (1.641, 1.652) among Whites only P for interaction < 0.0001. Similarly, aOR of the association of ETSE with depression was 1.772 (1.766, 1.779) among those who have never married only, 2.285 (2.278, 2.292) among married subjects only and 0.403 (0.400, 0.405) for those who are widowed P for interaction < 0.0001.

**Table 8:** Subgroup analyses of the association of ETSE with depression among non-smokers in the 2015–2016 NHANES Survey

Characteristics	OR (95%CI)	*P*	*P for Interaction*
Sex			
Male	0.674 (0.670, 0.677)	< 0.0001	< 0.0001
Female	1.992 (1.987, 1.997)	< 0.0001	
Age groups			
< 60 years	1.792 (1.787, 1.796)	< 0.0001	< 0.0001
≥ 60 years	1.066 (1.060, 1.071)	< 0.0001	
Race			
Hispanics only	1.410 (1.403, 1.417)	< 0.0001	< 0.0001
Whites only	1.646 (1.641, 1.652)	< 0.0001	
Blacks only	0.552 (0.549, 0.556)	< 0.0001	
Others	6.245 (6.208, 6.283)	< 0.0001	
Employed			
No	2.353 (2.346, 2.360)	< 0.0001	< 0.0001
Yes	1.123 (1.120, 1.127)	< 0.0001	
Annual Household Income			
*≤ $24,999*	1.289 (1.285, 1.294	< 0.0001	< 0.0001
*> $24,999*	1.795 (1.790, 1.800)	< 0.0001	
Marital Status			
Never married	1.772 (1.766, 1.779	< 0.0001	< 0.0001
Married	2.285 (2.278, 2.292)	< 0.0001	
Widowed	0.403 (0.400, 0.405)	< 0.0001	
Alcohol use			
No	1.470 (1.463, 1.477)	< 0.0001	< 0.0001
Yes	1.708 (1.704, 1.712)	< 0.0001	

*OR* odds ratio; *CI* confidence interval

## Discussion

The current study provides evidence for the association of ETSE with depression in the 2015-2016 NHANES data from the US. First, the rates of ETSE was relatively high. Second, depression was relatively prevalent. Third, females and young adults exposed to ETSE were at higher odds of being depressed. However, males exposed to ETSE were not at higher odds of being depressed.

Several reports [7, 12, 16, 36, 37] have attempted to present evidence on the burden of smoking in different populations with limited information on ETSE rates. In our study, we found about two in every ten non-smokers reported ETSE. Our findings on the burden of ETSE among non-smokers were comparable to a nationally representative tobacco survey among never-smoking youths from 168 countries between 1999 and 2008 [38]. In that study, about 23% of never-smoking youth were secondarily exposed to tobacco. In contrast, findings from a similar population in other climes revealed ETSE was as high as over 70% in China [39] and Spain [40]. Despite the recent decline in tobacco use, ETSE remains a potential threat to public health [41], and continuous efforts to reduce the burden of ETSE is vital. Globally, daily smoking rates appear lower, but the number of people using tobacco has increased [7]. In principle, passive exposure to tobacco smoke among non-smokers remains a public health threat in most regions of the world [42–45] and has been reported as a leading cause of death in the US [46]. However (in the light of our findings), evidence-based intervention efforts are necessary not only to meet reduction targets to manage the escalating burden of morbidity and mortality attributable to tobacco use but also ETSE. Also, more studies evaluating the potential contributions of ETSE to specific causes of morbidity/mortality are necessary.

In our study, the proportion of respondents with ETSE was significantly higher among young adults than old adults. Similarly, we found respondents exposed to ETSE at higher odds of being depressed in the overall population with aggravated odds among young adults and females. Our findings are in tandem with similar reports [47–54], where ETSE was observed to be associated with odds of psychological/mental distress. In contrast, other reports [47, 55, 56] found no significant association between ETSE and depression. These results can be explained in several ways.

First, ETSE is a proxy for discerning stressful living conditions [57, 58]. It may imply respondents exposed to ETSE might have been subjected to living and working conditions that predispose them to depression. In tandem with this assertion, lesser odds of depression and higher tendencies of a healthier lifestyle have been reported among persons from homes where smoking is prohibited [59]. Also, a diathesis-stress model has posited the association of ETSE with depression might be a function of stress [60]. Susceptibilities to the stressful environment (which may be evident by ETSE) may promote stress-induced depressive symptoms.

Second, the ETSE-depression relationship may be plausible. Using animal models, a neuro-biological route (via the dopamine complex assembly) has been reported for the causal association between ETSE and depression [25–29]. For example, caudate-putamen localization of dopamine D1 and D2 receptors was observed in rats with ETSE [27]. Similarly, alterations in the magnitude of dopamine D1 and γ-aminobutyric acid β2 receptors in the caudate-putamen may up-regulate dopamine transporter mRNA expression to promote neuropsychological disorders related to the midbrain abnormalities in rats [28]. Also, ETSE was observed to inhibit dopamine reuptake in the in-vivo [26], and low dopamine activity was associated with increased odds of major depressive disorders [61].

Contrary to the expectation, we found ETSE was inversely associated with depression among males. Our findings were in tandem with a report in Germany [55] highlighting a similar observation among males. On the one hand, suggesting high exposure to ETSE is likely to be associated with lower odds of being depressed (independent of sex, race and marital status) would be implausible. On the other hand, it is tedious to hypothesize that the latent noxious effect of ETSE on depression might be counteracted by an unknown factor(s) primarily related to social livelihood and systems patronizing males more than females. First, some reports [20, 55, 62] have suggested that females are likely to be vulnerable to ETSE from family members and colleagues who smoke at home and the workplace. Second, sex-related hormonal difference(s) that accompany mechanistic change(s) in the pathophysiology of diseases [63] is a plausible explanation for the difference in odds of depression between males and females in this study. In tandem with this postulation, gender-related differences have been reported in the onset of depression events in the entire life course [64]. Similarly, a correlation between the prevalence of depression and hormonal changes (during puberty, pregnancy, and perimenopause) has been hypothesized among females [65]. However, this observation does not connote the simplification of the potential effects of ETSE on the odds of being depressed among males. Future studies might consider discerning underlying genetic differences that confer different response(s) to depression in the light of ETSE.

Some limitations in this study are worth mentioning. The cross-sectional method excludes causal interpretations for the ETSE-depression relationship. ETSE was self-reported. It would be necessary to clarify the significance of the magnitude and duration of ETSE in future studies. The possibility of ETSE as an indicator for poor living conditions cannot be ruled out. Depression is likely to have been subjectively estimated given that it was self-reported and not a physician-administered assessment. Also, data on proximal drivers of depression (such as; living conditions, emotional stressors, coping and/or adapting mechanisms, etc.) were unaccounted for in our study. Howbeit, our data source and methodologies greatly enhanced the quality and reliability of our data. Hence, the main conclusions of this study remain valid and largely unaffected in the light of the large sample size, sampling strategy, statistical adjustment for potential confounding factors and weighting, which improved the statistical power of the study to be representative of the US population. Future longitudinal studies are essential to determine the causal association between ETSE and depression.

## Conclusion

Females exposed to ETSE (compared to those unexposed) were at higher odds of being depressed, but males exposed to ETSE were not at higher odds of being depressed. Intervention efforts targeted at policy formulation and behavioural change should be directed at tobacco control and the prevention of ETSE.

## Data Availability

Data for this study were sourced from the 2015–2016 National Health and Nutrition Examination Survey (NHANES) of the United States. The NHANES data was provided by the Center for Disease Control of the United States. It is open and publicly accessible through the following link; https://wwwn.cdc.gov/nchs/nhanes/.

## Abbreviations

aOR: Adjusted odds ratio
CDC: Centre for disease control and prevention
NHANES: National health and nutrition examination survey
ETSE: Environmental tobacco smoke exposure
US: United States
PHQ-9: Patient health questionnaire
